# Intra-arterial chemoradiotherapy for locally advanced oral cavity cancer: analysis of therapeutic results in134 cases

**DOI:** 10.1038/sj.bjc.6604272

**Published:** 2008-02-19

**Authors:** N Fuwa, T Kodaira, K Furutani, H Tachibana, T Nakamura, R Nakahara, T Tomoda, H Inokuchi, T Daimon

**Affiliations:** 1 Department of Radiation Oncology, Aichi Cancer Center Hospital, Nagoya, Japan; 2 Division of Drug Evaluation & Informatics, Graduate School of Pharmaceutical Sciences, University of Shizuoka, Shizuoka, Japan

**Keywords:** carcinoma of the oral cavity, intra-arterial chemotherapy, radiation therapy, prognostic factor

## Abstract

The objective of this study was to investigate the therapeutic results of arterial injection therapy via the superficial temporal artery for 134 cases of stages III and IV (M0) oral cavity cancer retrospectively, and to clarify the prognostic factors. We administered intra-arterial chemoradiotherapy by continuous infusion of carboplatin in 65 cases from January 1993 to July 2002. Systemic chemotherapy was performed on 26 cases at the same time. We administered intra-arterial chemoradiotherapy by cisplatin with sodium thiosulphate in 69 cases from October 2002 to December 2006. Systemic chemotherapy was performed on 48 cases at the same time. The 3-year local control rate was 68.6% (T2-3: 77.9%; T4: 51.3%), and the 3-year survival rate was 53.9% (stage III: 62.9%; stage IV: 45.3%). Regarding the results of multivariate analysis of survival rates, age (<65), selective intra-arterial infusion, and the use of cisplatin as an agent for intra-arterial infusion were significant factors. The therapeutic results of intra-arterial chemoradiotherapy via the superficial temporal artery were not inferior to the results of surgery. In particular, the results of arterial injection therapy by cisplatin with sodium thiosulphate were excellent, so we believe that it will be a new therapy for advanced oral cavity cancer.

As locally advanced oral cavity cancer is difficult to control by radiotherapy, surgery remains the most effective curative therapy (Poulsen *et al*, 1996). In this case, an extended surgery markedly reduces the quality of life, thus affecting the patient's social life. Therefore, the development of effective non-resection therapy is extremely important.

In 1992, we started chemoradiation therapy, in which continuous arterial infusion therapy with carboplatin was combined with radiotherapy, by selectively inserting a catheter into the target artery through the superficial temporal artery in patients with locally advanced head and neck cancer (Fuwa *et al*, 2000). We started therapy in two courses of systemic chemotherapy combined with intra-arterial chemoradiotherapy by continuous intra-arterial infusion of carboplatin and radiation therapy, in an effort to control metastasis in cervical lymph nodes and distant metastasis in 1997 (Fuwa *et al*, 2007). Furthermore, we changed the agent for intra-arterial infusion from carboplatin to cisplatin in an effort to improve the local control rate in October 2002. This is an improved technique of the Robbins *et al* method (Robbins *et al*, 1994, 2000), whereby an infusion dose of cisplatin was increased by infusing sodium thiosulphate, which is a neutralising agent of cisplatin, from a vein at the time of intra-arterial infusion of cisplatin.

In this article, we analyse the therapeutic results of 134 cases of stages III and IV (M0) oral cavity cancer retrospectively to investigate the prognostic factors, and we inspect the effectiveness of arterial injection therapy via the superficial temporal artery for cases of advanced oral cavity cancer.

## MATERIALS AND METHODS

### Patient selection criteria

The subjects met the following criteria: (1) the pathology is squamous cell carcinoma; (2) stage III or higher oral cavity cancer (except carcinoma of the base of tongue) without distant metastasis according to the TNM staging published in 2002; (3) patients in whom the performance status (PS) was evaluated as 0–3 according to the classification described by the Eastern Cooperative Oncology Group; (4) ages ranging from 20 to 89 years; (5) the bone marrow function was maintained (leukocyte count: 3000 mm^−2^ or more, platelet count: 100 000 mm^−2^ or more); (6) patients without severe liver, kidney, heart, or lung dysfunction; (7) untreated patients; (8) patients without active double cancer at the start of treatment, and who had not previously undergone radiotherapy in the head and neck region; and (9) patients from whom written informed consent was obtained.

### Treatment schedule, administration of the agent

**Figure 1:**
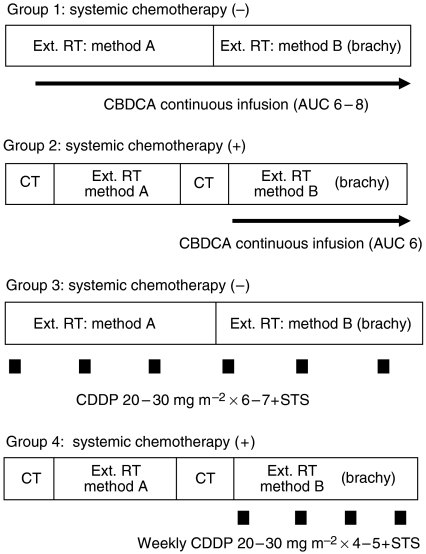
Scheme of the therapy. CT, chemotherapy; Ext RT, external beam radiation therapy. Method A: wide field irradiation, 36–40 Gy/20 fraction. Method B: reduced field irradiation, 26–30 Gy/15 fraction. Brachy, brachytherapy. Alternating therapy involving systemic chemotherapy and radiation therapy is performed. Intra-arterial chemotherapy is initiated after the end of the second course of systemic chemotherapy in the Groups 2 and 4.

The treatment schedule was divided into four groups (Figure 1). Continuous arterial injection of carboplatin was performed using a portable electrical pump for 6 weeks in Group 1. Using Calvert's formula, the total dose of carboplatin was established as six- to eight-fold the area under the plasma concentration–time curve (AUC) according to both the kidney function and PS.

As mentioned above, we started a new chemoradiation therapy in which continuous arterial infusion therapy with carboplatin was combined with two courses of systemic chemotherapy with 5-fluorouracil and nedaplatin in order to control the neck lymph nodes and distant metastases (Group 2) in 1997. Carboplatin (total dose: AUC 6) was administered continuously in the latter half of radiotherapy after the end of the second course of systemic chemotherapy. The regimen of systemic chemotherapy consisted of continuous intravenous injection of 5-fluorouracil at 700 mg m^−2^ for 5 days (from Day 1 until Day 5) and intravenous drip of nedaplatin at 120 mg m^−2^ over 6 h on Day 6.

To further improve the local control, we modified the procedure described by Robbins *et al* from October 2002. The dose of cisplatin was established as 20 mg m^−2^ when the catheter was inserted into the selected artery, and 30 mg m^−2^ when the catheter was inserted into the external carotid artery. During the arterial injection of cisplatin, a cisplatin-neutralising agent, sodium thiosulphate, at 8–10 g m^−2^ was intravenously administered over 7 h.

When inserting catheters into arteries on both sides, we set the amount of the infused dose of CDDP up to 40 mg m^−2^ in total per week; and to distribute the agent appropriately, we decided the amount of agent distributed from the findings of the MRI.

In patients who were not eligible for systemic chemotherapy, including elderly patients (⩾75 years) and those with a poor PS score, cisplatin arterial injection chemotherapy was repeated six to seven times in combination with radiotherapy at 60–70 Gy (Group 3).

In patients in whom systemic chemotherapy was possible, alternating therapy involving systemic chemotherapy and radiation therapy was performed. Arterial injection therapy was repeated four to five times after the end of the second course of systemic chemotherapy (Group 4). The regimen of systemic chemotherapy consisted of the continuous intravenous injection of 5-fluorouracil at 700 mg m^−2^ for 5 days (from Day 1 until Day 5) and intravenous drip of cisplatin at 85 mg m^−2^ over 24 h on Day 6. In patients with a poor renal function (24-h creatinine clearance was 60 ml min^−1^ or less), nedaplatin at 100 mg m^−2^ was administered in place of cisplatin.

### Radiation therapy

Radiotherapy was performed five times a week by irradiating 1.8–2 Gy of photon beam in a fraction using a 6 MV linear accelerator. The initial irradiation (irradiation method A) was performed five times a week for 4 weeks at a radiation dose of 1.8–2 Gy (total dose: 36–40 Gy). The latter half of irradiation (irradiation method B) was performed five times a week for 3 weeks at a radiation dose of 2 Gy (total dose: 26–30 Gy) (A+B: 66 Gy).

In the irradiation method A, using the bilateral opposing portal irradiation method, 36–40 Gy in 20 fractions was irradiated between the primary lesion, the middle cervical lymph nodes, and a 2 cm safety margin, whereas 36–40 Gy of photon beam was irradiated between the lower cervical region and the supraclavicular fossa using the anterior single irradiation method.

In irradiation method B, an area involving the tumour site on the initial consultation and a 1 cm safety margin was established as the planned target volume (PTV). The radiation dose for the spinal cord was established as 40 Gy or less. In patients with tongue or oral floor cancer in whom brachytherapy was possible, external irradiation at a radiation dose of approximately 50 Gy or less was combined with brachytherapy using a Cs needle or Au grain.

### Arterial injection therapy

As previously reported (Fuwa *et al*, 2000), the anterior ear on the affected side was incised under local anaesthesia to expose the superficial temporal artery. During fluoroscopy, a thin catheter was selectively inserted into the selected artery. When the lesion involved the contralateral side beyond the median line, another catheter was inserted in the contralateral side for bilateral arterial injection. The target artery was the lingual artery in carcinoma of the tongue, the facial artery in carcinomas of the floor of mouth, the buccal mucosa, and lower gingiva, and the maxillary artery in carcinomas of the hard palate and upper gingiva.

When the tumour involved beyond the perfusion area by selected arterial injection, or when severe arteriosclerosis made the selective insertion of a catheter into the selected artery difficult, a catheter was placed in the external carotid artery.

We confirmed that the extent of arterial injection covered the tumour by a pigment, angiography, and MRI from 2001, in which an extremely low dose of contrast medium for MRI was slowly infused via a catheter for arterial injection.

This clinical trial was approved by the Ethics Committee of Aichi Cancer Center Hospital.

### Patient assessments

The treatment response was evaluated based on the MRI. The subjects consulted the outpatient clinic at 1-month intervals for 1 year after the end of treatment, at 2- to 3-month intervals in the second and third years of follow-up, and at 3–5 month intervals after 3 years of follow-up. Follow-up MRI was performed at 4- to 6-month intervals for 2 years after the end of treatment, and at 6- to 8-month intervals thereafter. Chest X-rays were performed at 6- to 8-month intervals, and liver CT or echogram was performed every year until 3 years after the end of treatment.

Regarding the factors that affect the local control rate, we investigated the age (less than 65 years of age *vs* 65 years of age and over), the T stage (T2, T3 *vs* T4), the site (tongue *vs* oral cavity except tongue), the presence of systemic chemotherapy (Groups 1, 3 *vs* Groups 2, 4), the difference between selective intra-arterial infusion and non-selective intra-arterial infusion (external carotid artery), and the difference between agents for intra-arterial infusion (carboplatin *vs* cisplatin). Regarding the factors that affect the survival rate, we investigated N stage (N0, 1 *vs* N2, 3), clinical stage (III *vs* IV), and PS (0, 1 *vs* 2, 3) in addition to the six factors noted above.

We used the Kaplan–Meier method for survival and local recurrence-free analyses and the log-rank test to determine whether any significant differences existed between different patients in terms of end points. Survival and local recurrence-free rates were calculated (as of April 1, 2007 or the date of the last medical examination) from the start of treatment to the date of the event.

The Cox regression model was used to perform a multivariate analysis.

## RESULTS

### Patient population

The subjects consisted of 136 patients with locally advanced oral cavity cancer who underwent intra-arterial chemotherapy combined with radiation therapy between January 1993 and December 2006 (Table 1). Because the amount of agent for intra-arterial infusion in two cases out of 136 was less than 50% of the scheduled amount, we performed an analysis with 134 cases infused with 50% or more of the prescribed amount.

Table 2 shows the TNM staging, age, and PS among the four groups. The median age of Groups 1 and 3 was 17 years older than that of the Groups 2 and 4. The percentage of good PS patients was higher in the Groups 2 and 4. Written informed consent was obtained from all the patients.

Follow-up studies were sufficiently performed in the 131 patients except in three patients as of April 2007. The median follow-up duration for patients who were alive was 45.4 months (range: 5–168 months).

### Treatment delivery

The selected arteries consisted of the lingual artery in 52 patients, the bilateral lingual arteries in 12 patients, the facial artery in 12 patients, the faciolingual trunk in 4 patients, the maxillary artery in 1 patient, the external carotid artery in 48 patients, the external carotid artery and contralateral lingual artery in 3 patients, and the external carotid artery and contralateral facial artery in 1 patient. During the treatment course, the route was changed from the lingual artery to the external carotid artery in two patients. The total dose of carboplatin ranged from 240 to 800 mg, with a median of 430 mg. The total dose of cisplatin ranged from 40 to 390 mg, with a median of 120 mg. In the arterial injections of carboplatin, 87% of the cases were administered the scheduled quantities of carboplatin, whereas 5% of the cases were administered 50% or less of the scheduled quantities of carboplatin. In the arterial injections of cisplatin, 75% of the cases were administered the scheduled quantities of cisplatin, whereas 5% were administered 50% or less of the scheduled quantities of cisplatin.

Of the patients, 74 (55.2%) patients received systemic chemotherapy. The number of chemotherapy courses was one in 9 patients, and two in 65 (87.8%) patients.

The radiation dose ranged from 27 to 78 Gy, with a median of 63 Gy. Brachytherapy was performed in 41 (30.6%) patients; interstitial irradiation using a Cs needle was performed on 14 patients, and interstitial irradiation using Au grain was performed on 27 patients.

## TREATMENT RESULTS

**Figure 2:**
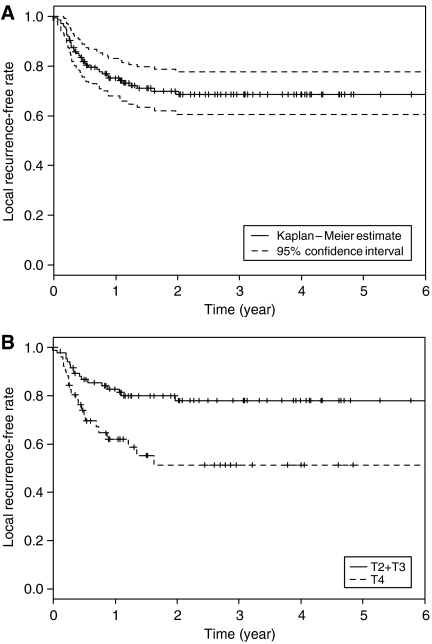
Actuarial local (primary site) recurrence-free rate of patients with advanced squamous cell carcinoma of the oral cavity by Kaplan–Meier method. (**A**) Actuarial local (primary site) recurrence-free rate of all patients. A solid line: local control rate curve. A broken line: 95% CI. (**B**) Actuarial local (primary site) recurrence-free rate according to the T stage. A solid line: T2+T3 cases (*n*=83). A broken line: T4 cases (*n*=51). *P*=0.00531.

A complete response was achieved in 109 patients, and a partial response in 25 patients. A relapse was detected in 65 patients: primary site, 36 patients; cervical lymph node, 19 patients; primary site and cervical lymph node, 2 patients; primary site and distant metastasis, 1 patient; cervical lymph node and distant metastasis, 2 patients; distant metastasis, 5 patients. The 3-year local (primary site) recurrence-free rate of all patients was 68.6% (95% confidence interval (CI): 60.6–77.7%) (Figure 2A). Cumulative local recurrence-free rate of T2-3 and T4 patients at 3 years were 77.9% (95% CI: 69.1–87.9%) and 51.3% (95% CI: 37.5–70.2%), respectively (Figure 2B).

Of the patients demonstrating a relapse, salvage surgery was performed in 15 patients, intra-arterial chemoradiation in 9 patients, intra-arterial chemotherapy in 3 patients, chemoradiation therapy in 3 patients, radiation therapy in 5 patients, and chemotherapy in 1 patient. Of these 36 patients, 9 patients had successful salvage (surgery in 5 patients, intra-arterial chemoradiation in 3 patients, chemoradiation therapy in 1 patient), becoming disease-free after the procedure.

**Figure 3:**
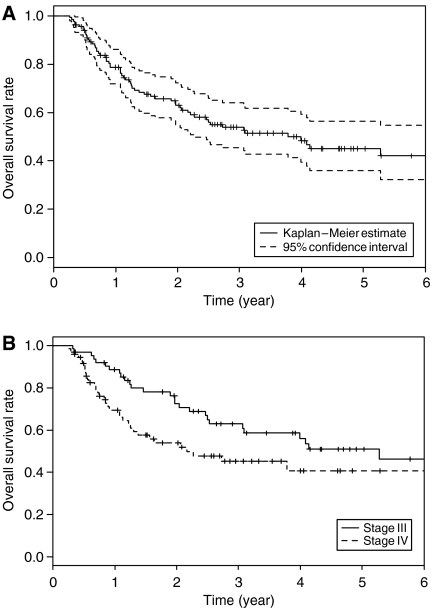
Actuarial survival rates of patients with advanced squamous cell carcinoma of the oral cavity by Kaplan–Meier method. (**A**) Actuarial survival rates in all 134 patients. A solid line: overall survival curve. A broken line: 95% CI. (**B**) Actuarial survival rates according to the stage. A solid line: stage III cases (*n*=63). A broken line: stage IV cases (*n*=71). *P*=0.117.

At the time of analysis, 65 patients had died, 66 patients were still alive, and 3 patients had been lost to the follow-up. In the 65 patients who had died, the cause of death was oral cavity cancer in 45, other diseases in 18, and treatment-related complication in 2. The 3-year overall survival of all patients was 53.9% (95% CI: 45.4–64.0%) (Figure 3A). Cumulative survival rates of stages III and IV patients at 3 years were 62.9% (95% CI: 51.4–77.0%) and 45.3% (95% CI: 33.9–60.5%), respectively (Figure 3B).

### Factors of survival and local recurrence

In a univariate analysis, T factor, the selected artery, and the site were found to have a significant impact on local recurrence, whereas systemic chemotherapy and difference of IA chemotherapy had only a marginal significance. In a multivariate analysis, T factor and difference of IA chemotherapy were of borderline significance (Table 3).

In a univariate analysis, age, systemic chemotherapy, and difference of IA chemotherapy were found to have a significant impact on survival. In a multivariate analysis, age, difference of IA chemotherapy, and selected artery were found to have a significant impact on survival, whereas systemic chemotherapy was not a significant factor (Table 4).

### Acute toxicity

Acute toxicity is summarised in Table 5. Grade 3 or higher toxic changes included granulocytopaenia in 60 (45%) patients, thrombopaenia in 31 (23%) patients, anaemia in 26 (19%) patients, and mucositis in 15 (11%) patients. There was no significant difference in the degree of acute toxicity among the four groups.

In addition, no transient or persistent central nervous complications were observed. Treatment-related death was confirmed in two patients. Although tumours in both patients disappeared as a result of therapy, both patients died of gastrointestinal bleeding.

### Chronic toxicity

We studied chronic toxicity in 97 patients who survived more than 12 months after the treatment. Although these 97 patients did not develop severe problems in their phonation or deglutition function and were able to eat almost normally, continuous glossalgia was recognised in one of the patients and analgesic was sometimes necessary for this patient, and two patients developed osteo-radionecrosis, which needed surgery.

## DISCUSSION

The results of radiotherapy for advanced oral cavity cancer alone were poor (Decroix and Ghossein, 1981; Horiuchi *et al*, 1982). Currently, surgery is the standard treatment (Poulsen *et al*, 1996). Several studies have reported arterial injection therapy for oral cavity cancer; however, the number of patients in such studies tended to be small, and its usefulness has not yet been clearly demonstrated (Hirai *et al*, 1999; Damascelli *et al*, 2003; Kovacs, 2004). No randomised controlled trials have yet been performed to compare the effectiveness of surgery with chemoradiation, and the usefulness of chemoradiation therapy involving systemic chemotherapy thus remains to be clarified.

After surgery, the 5-year survival rate ranged from 27 to 60% of stage III patients, while it ranged from approximately 12 to 40% of stage IV patients (Chen *et al*, 1999; Sessions *et al*, 2002; Greenberg *et al*, 2003; Lo *et al*, 2003; Gorsky *et al*, 2004; Liao *et al*, 2006; Fan *et al*, 2007); our results were therefore similar to the results after surgery. In particular, the results of arterial injection therapy by cisplatin with sodium thiosulphate were excellent.

Currently, there are two procedures for performing arterial infusion therapy for head and neck cancer: namely, a procedure in which a catheter is inserted into the target artery through the superficial temporal artery, as presented in this study, and a procedure in which a catheter is inserted into the target artery through the femoral artery by Seldginger's procedure (Robbins *et al*, 1994, 2000; Balm *et al*, 2004; Alkureishi *et al*, 2006). The latter procedure is simpler than the former procedure, and it facilitates the administration of anticancer agents into several arteries; however, drug administration over a long duration is impossible, and catheter operation-related cranial nerve disorders may sometimes occur. Although Robbins *et al* reported the incidence of cranial nerve disorders to be 2–4%, no patient showed a cranial nerve disorder in our study. While, in his series, the mean age was 56 years, the median age was 67 years in our study. Therefore, arterial injection therapy in which a catheter is inserted into the target artery through the superficial temporal artery may therefore be appropriate for elderly patients, and the drug can thus be administered over a long duration.

The dose of cisplatin in our study was approximately 1/5 of that described by Robbins *et al*. However, the duration of administration was 60 times longer. It has been reported that the antitumour effects of cisplatin are correlated with the concentration and duration of administration (DeConti *et al*, 1973; Drewinko *et al*, 1973); low-dose cisplatin may exhibit potent antitumour effects.

It remains unclear whether a neutralizer sodium thiosulphate needs to be added to such administered quantities, but we believed that the addition of sodium thiosulphate was necessary, because our study involved elderly patients and patients who have malfunctioning kidneys, and also because the combined usage of systemic chemotherapy was one of the assumptions of the study. Regarding the radiation dosage when arterial injection chemotherapy and radiotherapy were used concomitantly, we administered the same doses as those that we used for cases of combined use of systemic chemotherapy. One of our future tasks is to examine long-term adverse effects, but no severe late-onset effects have been observed thus far.

A randomised controlled trial was performed in the Netherlands, comparing chemoradiation that used cisplatin as a drug agent for arterial injection in a method similar to that of Robbins *et al* with chemoradiation that used cisplatin as systemic chemotherapy. Unfortunately, the initial outcome showed no difference between the survival rates of the therapies. In the Netherlands study, there were few oral cavity cancer patients (Balm and Rasch, 2006). Although the effectiveness of chemoradiation that concomitantly uses systemic chemotherapy has been clarified for cases of pharyngeal and laryngeal cancer, it has not yet been clarified for cases of oral cavity cancer. Thus, we suggest that randomised controlled trials limit their target cases to, for example, patients with oral cavity cancer.

The results of our study were similar to those of surgery, thus suggesting the usefulness of arterial injection therapy combined with radiation therapy. In particular, the results of arterial injection therapy by cisplatin with sodium thiosulphate were excellent, so we believe that it will be a new therapy for locally advanced oral cavity cancer.

## Figures and Tables

**Table 1:** Characteristics of 134 patients with squamous cell carcinoma of the oral cavity

*Age (years)*	
Median	67
Range	25–89
	
*Gender*	
Male	89
Female	45
	
*Performance status (ECOG)*	
0	26
1	93
2	11
3	4
	
*TNM (2002) T stage*	
T1	0
T2	16
T3	67
T4a	49
T4b	2
	
*TNM (2002) N stage*	
N0	61
N1	34
N2a	1
N2b	28
N2c	6
N3	4
	
*Stage*	
III	63
IV A	67
IV B	4
	
*Primary tumour site*	
Tongue	88
Lower gingiva	16
Floor of the mouth	14
Buccal mucosa	12
Upper gingiva	3
Hard palate	1
	
*Reasons not performing surgery*	
Refusal	78
Old age	27
Poor performance status	10
Poor cardio-pulmonary function	10
Inoperable advanced lesion	9

**Table 2:** TNM stage, age, and PS among the groups

	**Group 1 (*n*=39)**	**Group 2 (*n*=26)**	**Group 3 (*n*=21)**	**Group 4 (*n*=48)**
*Stage*				
III	26	14	5	18
IVA	13	12	14	28
IVB	0	0	2	2
				
*Age (years)*				
Median	73	59	77	59
Range	51–90	25–73	62–87	25–73
				
*PS*				
0	2	8	3	13
1	29	17	14	33
2	6	1	3	1
3	2	0	1	1

**Table 3:** Results of the multivariate analysis of prognostic factor on local recurrence-free time based on Cox proportional-hazards model

**Selected factor**	**Level**	**Adjusted *P*-value^a^**	**Adjusted HR (95% confidence interval)**
T classification	T2 or T3	0.0501	1.000 (referent)
	T4		2.11 (0.999, 4.45)
			
Site	Tongue	0.684	1.000 (referent)
	Oral cavity except tongue		1.16 (0.558, 2.42)
			
Systemic chemotherapy	NO (Group 1 or 3)	0.265	1.000 (referent)
	YES (Group 2 or 4)		0.682 (0.347, 1.33)
			
IA chemotherapy	CBDCA	0.0884	1.000 (referent)
	CDDP		0.552 (0.278, 1.09)
			
Artery	Selected artery	0.106	1.000 (referent)
	External carotid artery		1.76 (0.885, 3.53)

a The *P*-value for the log-rank test.

**Table 4:** Results of the multivariate analysis of prognostic factors on overall survival based on Cox proportional-hazards model

**Selected factor**	**Level**	**Adjusted *P*-value)^a^**	**Adjusted HR (95% confidence interval)**
Age (year)	<65	0.0185^*^	1.000 (referent)
	>=65		2.05 (1.12, 3.75)
			
Systemic chemotherapy	NO (Group 1 or 3)	0.104	1.000 (referent)
	YES (Group 2 or 4)		0.610 (0.336, 1.10)
			
IA chemotherapy	CBDCA	0.0141^*^	1.000 (referent)
	CDDP		0.477 (0.265, 0.861)
			
Artery	Selected artery	0.0290^*^	1.000 (referent)
	External carotid artery		1.74 (1.05, 2.86)

a The *P*-value for the Wald's test.

^*^P-value<0.05.

**Table 5:** Toxicity according to the group

	**Group 1 (*n*=39)**			**Group 2 (*n*=26)**			**Group 3 (*n*=21)**			**Group 4 (*n*=48)**		
Toxicities	0–2	3	4	0–2	3	4	0–2	3	4	0–2	3	4
*Haematologic*												
White blood cell	21	15	3	15	5	6	9	8	4	24	18	6
Granulocyte	22	13	4	15	5	6	11	8	2	26	14	8
Platelet	32	4	3	14	6	6	18	2	1	39	4	5
Haemoglobin	34	4	1	20	6	0	18	3	0	36	10	2
												
*Non-haematologic*												
Liver	39	0	0	25	1	0	21	0	0	47	1	0
Kidney	39	0	0	26	0	0	21	0	0	48	0	0
Vomiting	39	0	0	26	0	0	21	0	0	46	2	0
Mucocitis	37	2	0	23	3	0	19	2	0	40	8	0
Fever	37	2	0	25	1	0	20	1	0	48	0	0
